# Angiotensin II Disrupts Axo-Axonal Interaction-Mediated Vasorelaxation in Basilar Arteries of Normotensive and Hypertensive Rats

**DOI:** 10.3390/biomedicines14040853

**Published:** 2026-04-08

**Authors:** Stephen Shei-Dei Yang, Kuan-Yu Chen, Earl Fu, Hsi-Hsien Chang, Kuo-Feng Huang

**Affiliations:** 1 Division of Urology, Department of Surgery, Taipei Tzu Chi Hospital, Buddhist Tzu Chi Medical Foundation, New Taipei 231016, Taiwan; uroyang@gmail.com (S.S.-D.Y.); chsihsien@gmail.com (H.-H.C.); 2 New Taipei City Hospital, New Taipei 241204, Taiwan; kuanyu8514498000@yahoo.com.tw; 3 Department of Dentistry, Taipei Tzu Chi Hospital, Buddhist Tzu Chi Medical Foundation, New Taipei 231016, Taiwan; fuearl@gmail.com; 4 Division of Neurosurgery, Department of Surgery, Taipei Tzu Chi Hospital, Buddhist Tzu Chi Medical Foundation, New Taipei 231016, Taiwan; 5 School of Medicine, Tzu Chi University, Hualien 97004, Taiwan

**Keywords:** angiotensin II, ferroptosis, neurogenic vasodilation, basilar artery, sympathetic-parasympathetic nerve interaction

## Abstract

**Background/Objectives:** The renin–angiotensin–aldosterone (RAA) system is a key regulator of cardiovascular homeostasis. Recent evidence suggests that Angiotensin II (Ang II) can trigger ferroptosis, an iron-dependent form of cell death. We previously demonstrated that periodontitis induces neurovascular dysfunction, and our preliminary observations indicate that this oral inflammatory model is associated with elevated blood pressure. However, the mechanism by which Ang II impaired nitrergic vasodilation and triggered ferroptosis in cerebral arteries remains unclear. This study investigates the functional effects of electrical and chemical nerve stimulation in adult spontaneously hypertensive rats (SHR) and Wistar-Kyoto rats (WKY). **Methods:** Endothelium-denuded basilar arterial (BA) rings from SHRs and WKYs were used to assess the impact of Ang II on neurogenic relaxation via wire myography. **Results:** Vascular relaxation responses to nicotine and transmural nerve stimulation (TNS) were significantly diminished in SHRs compared to WKYs. This impairment was reversed by both acute preincubation and chronic treatment with losartan (an AT1 receptor antagonist). In WKY BAs, exogenous Ang II pretreatment inhibited relaxation responses to nicotine, TNS, and isoproterenol. Importantly, this inhibition was effectively reversed by marimastat (MMP inhibitor), catalase (antioxidant), and ferrostatin-1 (ferroptosis inhibitor). **Conclusions:** Our findings indicate that Ang II induces functional alterations in neurovascular signaling patterns by triggering ferroptosis within nerve terminals. This process leads to a functional imbalance between sympathetic and parasympathetic influences, ultimately impairing neurogenic nitrergic dilation in the BAs of SHRs. These results suggest that targeting Ang II-induced ferroptosis may alleviate the neuroinflammation and cognitive decline associated with hypertension-related cerebrovascular dysfunction.

## 1. Introduction

Our previous research demonstrated that silk ligation around molars induces a chronic oral inflammatory state associated with impaired neurovascular coupling of the cerebral basilar artery (BA) and elevated beta-amyloid (Aβ) expression [1]. We specifically found that Aβ inhibits nicotine-induced neurogenic vasorelaxation in the BA. Furthermore, our preliminary observations indicate that this oral inflammatory model is associated with elevated blood pressure. At the same time, proteomics analysis reveals a significant up-regulation of the angiotensin-activated signaling pathway in the rat brain.

The RAA system is a primary regulator of cardiovascular homeostasis [2]; its dysregulation is central to the pathogenesis of hypertension. In the cerebral region, levels of Ang II, a key RAA effector and neuropeptide, are significantly elevated in the brains of SHRs and renovascular hypertensive rats (RHRs) [3,4,5]. Most adverse vascular effects of Ang II are mediated via Ang II type 1 (AT1) receptors [6]. Activation of the cerebrovascular RAA impairs endothelial nitric oxide-dependent signaling, contributing to vascular dysfunction [7]. Our previous studies demonstrated that sympathetic-parasympathetic nerve interactions (axo-axonal interactions) normally enhance cerebral neurogenic nitrergic vasodilation and basilar arterial blood flow (BABF); however, this vasodilation mechanism was abolished in adult SHRs and RHRs [8,9].

Central to this dysfunction is Ang II, which impairs neurovascular coupling and increases reactive oxygen species (ROS) via NADPH oxidase [10]. Recent evidence also suggests that Ang II can induce ferroptosis, an iron-dependent form of regulated cell death driven by phospholipid peroxidation within the cerebral cortex [11,12,13,14]. Since ferroptosis is implicated in both neuronal injury and vascular smooth muscle damage, it may represent a critical pathway through which Ang II exacerbates neurodegenerative decline.

To isolate the systemic effects of the RAA system from confounding chronic inflammatory factors, the current study utilized genetically hypertensive SHRs. Although the RAA is increasingly linked to neurodegenerative disorders like Alzheimer’s disease [15,16], it remains unclear whether Ang II, through these multifaceted actions, directly reduces nitrergic vasodilation in the cerebral arteries. Therefore, we investigated the effects of electrical and chemical sympathetic nerve stimulation in adult SHRs and WKYs to elucidate the role of the Ang II/AT1 axis in mediating neurogenic vascular deficits.

## 2. Materials and Methods

All animal protocols were approved by the Laboratory Animal Care and Use Committee at Taipei Tzu Chi Hospital in accordance with guidelines for the care and use of laboratory animals. Male WKYs and SHRs (15–20 weeks old) were housed under controlled conditions, including a 12 h light/dark cycle (7:00 AM to 7:00 PM) and a temperature range of 21 °C to 23 °C.

### 2.1. Chronic Treatment with Losartan

Adult SHRs were used. Systolic blood pressure (SBP) was measured three times weekly using the tail-cuff method (BP Monitor Model MK-200, Muromachi Kikai Co., Ltd., Tokyo, Japan). Following a 7-day quarantine, losartan (10 mg/kg/day, oral) was administered to SHRs for 30 days, dissolved in 5 mL of double-distilled water (10 mg/mL). After treatment, the SHRs were euthanized for in vitro tissue bath studies.

### 2.2. In Vitro Tissue BATH Studies

WKYs and SHRs were anesthetized with zoletil (40 mg/kg, i.p.) and xylazine (10 mg/kg, i.p.). The entire brain, with dura mater attached, was removed and placed in Krebs bicarbonate solution (4 °C, equilibrated with 95% O_2_/5% CO_2_). The BA was dissected, cleaned of surrounding tissue under a dissecting microscope, and a 4 mm segment was isolated. The basilar arterial ring was mounted on two stainless steel wires (40 μm diameter) in a single-channel myograph (Radnoti M1 series, Monrovia, CA, USA) to measure isometric tension. The Krebs bicarbonate solution (37 °C, 95% O_2_/5% CO_2_) contained (in mM): NaCl 144.2, NaHCO_3_ 25, KCl 4.97, CaCl_2_ 1.6, MgSO_4_ 1.2, glucose 11.1, ascorbic acid 0.28, and calcium disodium EDTA 0.023. Tissues were equilibrated for 45 min, then stretched to a resting tension of 5 mN. All experiments used endothelium-denuded basilar arterial rings, confirmed by the absence of relaxation to acetylcholine (1 μM).

Step 1: Rings were pre-contracted with U-46619 (0.1 μM), followed by relaxation with transmural nerve stimulation (TNS, 10 ms, 4 or 8 Hz, 10 V, 20 s), nicotine (50 μM), norepinephrine (0.1, 1 μM), or isoproterenol (0.01–10 μM). Isometric force changes were recorded (PowerLab 8/30, LabChart 7). A 45 min washout followed.

Step 2: Losartan (1 μM) was preincubated for 15 min before repeating U-46619-induced contraction and relaxation with nicotine, norepinephrine, or isoproterenol. A 45 min washout followed.

Step 3: Step 1 was repeated (washout state).

To enhance ROS activation of matrix metalloproteinases (MMPs), basilar arterial rings in Step 2 were incubated in Krebs solution (37 °C) with Ang II (10 or 100 nM) for 60 min. Pre-contraction with U-46619 (0.1 μM) was performed, followed by TNS-, nicotine-, or isoproterenol-induced relaxation. After a 45 min washout, rings were preincubated for 30 min with Ang II (100 nM) combined with catalase (100 U/mL), marimastat (10 μM), or ferrostatin-1 (10 μM), followed by U-46619 pre-contraction and relaxation with TNS, nicotine, or isoproterenol.

Step 4: After a 45 min washout, Step 1 was repeated (washout state). Tension changes were expressed as a percentage of sodium nitroprusside (SNP, 0.1 mM)-induced maximum relaxation.

### 2.3. Drugs Used

The following chemicals were used: NaCl, NaHCO_3_, KCl, CaCl_2_, MgCl_2_, EDTA, ascorbic acid, and glucose (Amresco, Solon, OH, USA); sodium nitroprusside, nicotine, urethane, chloralose, acetylcholine, norepinephrine, angiotensin II, U-46619, isoproterenol, losartan, marimastat, catalase, ferrostatin-1, U-46619 and calcium disodium EDTA (all from Sigma-Aldrich, St. Louis, MO, USA).

### 2.4. Statistical Analysis

All data are presented as mean ± standard deviation and visualized using bar charts. Differences between the groups were analyzed using analysis of variance (ANOVA), followed by Bonferroni post hoc tests for normally distributed data. A *t*-test was performed to compare the specific differences between WKYs and SHRs. Statistical significance was defined as a *p*-value < 0.05. All statistical analyses were conducted using SigmaStat version 3.5 for Windows (Systat Software, San Jose, CA, USA).

## 3. Results

### 3.1. Nicotine- and TNS-Induced Relaxation of Basilar Arterial Rings in SHRs

**Figure 1:**
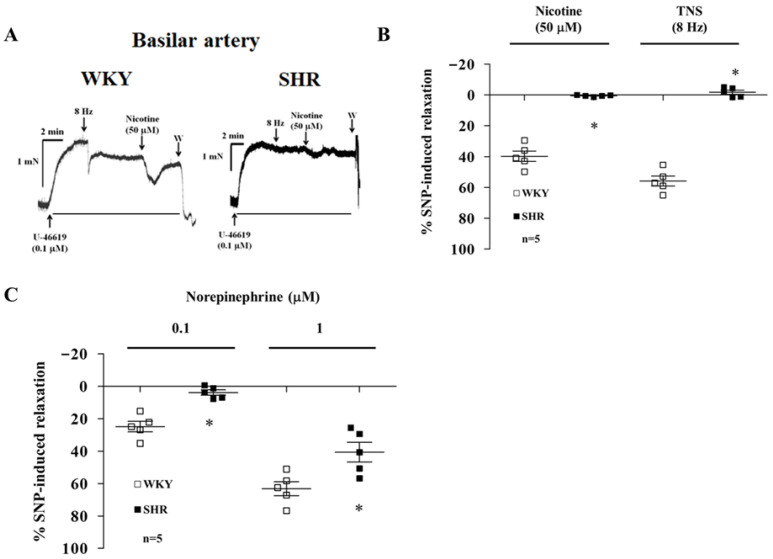
Representative tracings of nicotine- (50 uM) and TNS- (8 Hz) induced relaxation in endothelium-denuded basilar arterial rings from 20-week-old WKYs and SHRs. (**A**) Absence of vasorelaxation in SHRs, with summarized data in (**B**), *p*-values = 0.002. (**C**) Concentration-dependent relaxation to norepinephrine (NE, 0.1–1 uM) following U-46619 (0.1 uM) pre-contraction. NE-induced relaxation was significantly attenuated in SHRs. Data mean ± SD; n = number of experiments. (* *p* < 0.05) vs. WKYs (Student’s *t*-test).

In the presence of active muscle tone induced by U-46619 (0.1 μM), relaxation of endothelium-denuded basilar arterial rings was caused by nicotine (50 μM) and TNS (8 Hz) in WKYs (n = 5, Figure 1A,B). These relaxations were significantly diminished in the SHRs (n = 5, *p* < 0.05, Figure 1A,B).

### 3.2. Norepinephrine-Induced Relaxation of Basilar Arterial Rings in SHRs

Endothelium-denuded basilar arterial rings of WKYs were contracted by pre-treatment with U-46619, and relaxation was induced by treatment with norepinephrine (0.1 and 1 μM, n = 5, Figure 1C). This norepinephrine-induced relaxation was decreased in the SHRs (n = 5, *p* < 0.05, Figure 1C).

### 3.3. Effect of Losartan on Basilar Arteries of SHR

**Figure 2:**
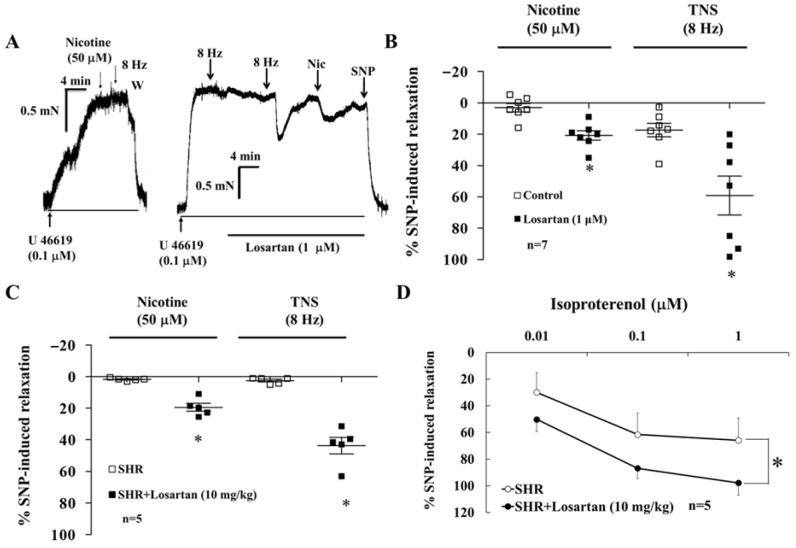
Representative tracing showing that the recovery of nicotine (50 μM)- and TNS (8 Hz)-induced endothelium-denuded relaxation of the basilar arterial ring in SHRs was significantly enhanced by pre-incubation with losartan (1 μM) for 15 min (**A**). These results are summarized in (**B**). Nicotine (50 μM)-, TNS-, and isoproterenol-induced vasodilation in the BA was significantly enhanced by oral losartan (10 mg/day) for 30 days (**C**,**D**). Values are mean ± SD. n = number of experiments. * *p* < 0.05 vs. Control. Asterisks indicate a significant difference in the *t*-test (* *p* < 0.05) vs. the control group in (**B**,**C**). Asterisks indicate a significant difference in Bonferroni post-tests following ANOVA (* *p* < 0.05) vs. SHR in (**D**).

In the presence of active muscle tone induced by U-46619 (0.1 μM), nicotine (50 μM)- and TNS (8 Hz)-induced relaxation of endothelium-denuded basilar arterial rings was diminished in SHRs (SYS/199.4 ± 6.2 mmHg, 20 weeks old, n = 15; Figure S1). The reduction in nicotine- and TNS-induced relaxation was significantly recovered by pre-incubation with losartan (1 μM) for 15 min (n = 7, *p* < 0.05, Figure 2A,B), and this failure was significantly recovered after oral losartan treatment (10 mg/kg/day/30 days) in SHRs (SYS/160 ± 2.9 mmHg, 20–21 weeks old, n = 5, *p* < 0.05, Figure 2C).

### 3.4. β-Adrenoceptor Agonist Isoproterenol-Induced Relaxation of Basilar Arterial Rings in SHRs

Endothelium-denuded basilar arterial rings were contracted by pre-treatment with U-46619, and relaxation was induced by treatment with isoproterenol (0.01, 0.1, and 1 μM). However, isoproterenol-induced relaxation was reduced in SHRs (n = 5, *p* < 0.05, Figure 2D). This isoproterenol-induced relaxation recovered after chronic losartan (10 mg/kg) treatment in SHRs (n = 5, *p* < 0.05, Figure 2D).

### 3.5. Ang II Inhibited Nicotine- and TNS-Induced Relaxation of Basilar Arteries in WKYs

**Figure 3:**
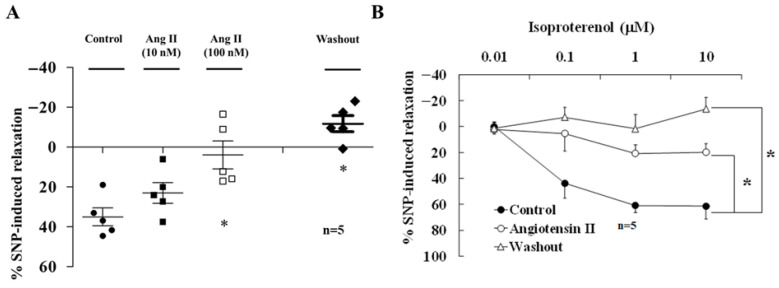
(**A**) Nicotine (50 μM)-induced relaxation of the endothelium-denuded basilar arterial rings of WKYs (15 weeks old) was inhibited by pre-treatment with AII (10–100 nM) in a concentration-dependent manner. (**B**) Isoproterenol (n = 5)-induced relaxation of the BA was significantly inhibited by AII (100 nM). The inhibition of AII was not recovered after AII washout. Values are mean ± SD. n = number of experiments. * *p* < 0.05 vs. Control. Asterisk indicates a significant difference in Bonferroni post-tests following ANOVA (* *p* < 0.05) vs. Control group.

In the presence of active muscle tone, nicotine (50 μM)-induced relaxation of endothelium-denuded basilar arterial rings in WKYs (15–20 weeks old) was significantly inhibited by pre-treatment with 100 nM Ang II (n = 5, *p* < 0.05, Figure 3A) for 60 min, in a concentration-dependent manner. ANG II inhibition did not recover after Ang II washout (n = 5; Figure 3A).

In the presence of active muscle tone, endothelium-denuded basilar arteries in WKYs were relaxed by administration of isoproterenol (0.01, 0.1, 1, and 10 μM) in a concentration-dependent manner (n = 5, Figure 3B). This relaxation was significantly inhibited by pre-treatment with 100 nM Ang II for 60 min (n = 5, *p* < 0.05, Figure 3B). Isoproterenol-induced relaxation did not recover after Ang II washout (n = 5, *p* < 0.05; Figure 3B).

### 3.6. Effects of Marimastat, Catalase, and Ferrostatin-1 on Ang II-Mediated Inhibition

**Figure 4:**
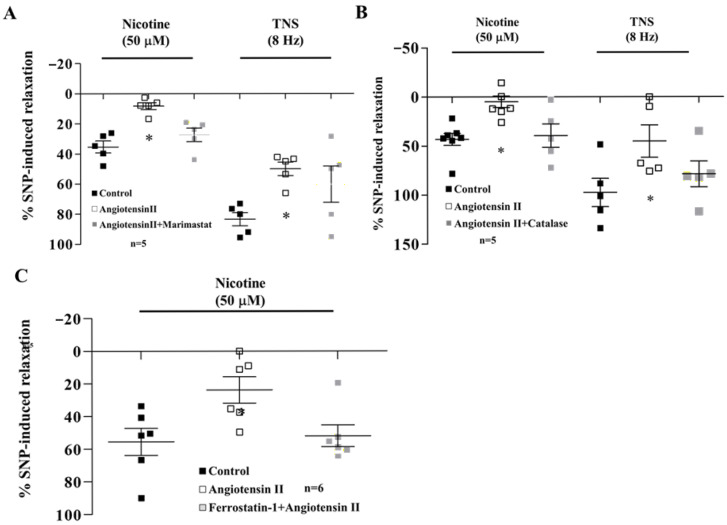
(**A**) Nicotine (50 μM)- and TNS (8 Hz)-induced relaxation was inhibited by pre-treatment with AII (100 nM), but this was significantly recovered by marimastat (1 μM). (**B**) Nicotine (50 μM)- and TNS (8 Hz)-induced relaxation was inhibited by pre-treatment with AII (100 nM), which was significantly recovered by catalase (100 U/mL). (**C**) The inhibition of AII (100 nM) was also recovered by ferrostatin-1 (10 μM). Values are mean ± SD. n = number of experiments. * *p* < 0.05 vs. Control. Asterisk indicates a significant difference in Bonferroni post-tests following ANOVA (* *p* < 0.05) vs. Control group.

In the presence of active muscle tone, nicotine-induced relaxation was significantly inhibited by pre-treatment with 100 nM Ang II (n = 5, *p* < 0.05). This inhibitory effect was significantly reversed by pretreatment of marimastat (10 uM, n = 5, *p* < 0.05, Figure 4A), catalase (100 U/mL, n = 5, *p* < 0.05, Figure 4C), or ferrostatin-1 (10 uM, n = 5, *p* < 0.05, Figure 4C).

## 4. Discussion

The current study demonstrated that SHRs have significantly diminished functional sympathetic-parasympathetic interactions for sympathetic nerve-elicited nitrergic vasodilation. The isolated endothelium-denuded BAs of 15–20-week-old SHRs did not relax following the application of nicotine or TNS. However, neurogenic relaxation was recovered following both chronic losartan treatment and acute losartan incubation. This indicates that the neurogenic nittorergic dilation of BAs, mediated by axo-axonal interactions, was inhibited in the presence of Ang II. This inhibition occurred via the production of ROS, with elevated MMP activity further contributing to the effect. These findings suggest that the persistent production of ROS and the increased MMP activity are mediated through the AT1 receptor. Consequently, these mechanisms may have induced ferroptosis in the BAs. Extending this to the broader mechanistic context, Ang II-induced dysfunction in cerebral and ocular vasculature is primarily driven by Nox2-dependent ROS formation rather than Nox1. However, caution is warranted in interpreting enzymatic sources; conventional NADPH-stimulated chemiluminescence assays may lack the specificity to accurately reflect Nox activity, as these signals can be confounded by nitric oxide synthase or cytochrome P450.

The differential distribution of Nox isoforms may further explain the selective vulnerability of the neurogenic pathway in our model. While Nox1 is a major contributor to ROS-mediated VSMC dysfunction in SHRs [17], Nox2 is the predominant isoform expressed in the adventitial layer, where perivascular nerve terminals reside [18]. Our findings suggest that Ang II-induced, Nox2-derived ROS production within the adventitia may specifically trigger lipid peroxidation and ferroptosis within the nitrergic terminals, thereby impairing neurogenic dilation while leaving the intrinsic smooth muscle relaxant machinery (as evidenced by the preserved isoproterenol responses) relatively operational. Although we did not utilize specific NADPH oxidase inhibitors such as VAS2870 in the current study, the potent restoration of nitrergic relaxation by Ferrostatin-1 provides strong functional evidence for the involvement of the NOX/ROS/ferroptosis axis in these neural components.

This approach is further supported by our pharmacological validation: the nicotine-induced increase in basilar arterial blood flow (BABF) was markedly blunted by the selective nNOS inhibitor 7-Nitroindazole [8,19]. Furthermore, the successful removal of the endothelium was verified by the complete absence of vasorelaxation in response to acetylcholine (ACh). These findings collectively confirm that the observed vasodilation is predominantly neurogenic, effectively eliminating confounding eNOS-derived signals to focus specifically on sympathetic-parasympathetic interactions.

Nicotine- or TNS-induced vasorelaxation was diminished in the SHRs’ basilar arterial rings (Figure 1A,B) compared with that of age-matched WKYs, and norepinephrine-induced vasorelaxation also decreased in the basilar arterial rings of SHRs (Figure 1C,D). Our previous study demonstrated that β_2_-adrenoceptors on cerebral perivascular parasynthetic nitrergic nerve terminals, which were mediated by electrical stimulation and nicotine activation of sympathetic nerve terminals, significantly increased the basilar arterial blood flow (BABF) [8]. However, β_2_-adrenoceptors in the central nervous system and autonomic nerve terminals are fully functional in early and established hypertension in similar-aged SHRs [20,21]. In addition, vascular smooth muscle β_1_ and β_2_-adrenoceptor-mediated isoprenaline-induced endothelium-independent vasorelaxation is impaired in SHRs [22], pre-hypertensive SHRs [23], and deoxycorticosterone-salt hypertensive rats [24]. These results suggest that the BAs of SHRs are characterized by a reduction in perivascular parasympathetic nerve terminals and impaired smooth muscle β-adrenoceptor function.

Treatment with losartan, administered either via preincubation (Figure 2A,B) or oral dosing (Figure 2C,D), yielded significant protective effects by restoring the diminished nicotine-, TNS-, and isoproterenol-induced vasorelaxation. Losartan has been reported to significantly reduce systemic blood pressure, decrease Ang II-induced increase in ROS, increase neuronal nitric oxide synthase phosphorylation, normalize sympathetic hyperreactivity, and restore β-adrenergic signaling pathway sensitivity [25]. Notably, while Aliskiren (a direct renin inhibitor) and losartan exhibited similar antihypertensive effects, only losartan prevented the activation of vascular profibrotic mechanisms and the upregulation of MMPs in 2K1C RHRs [26]. Ang II inhibited isoproterenol-induced BA relaxation; this inhibition persisted even after Ang II washout. However, treatment with losartan successfully restored this relaxant response. MMPs were directly involved in the degradation of existing β_2_- adrenoceptors on the cell surface; they did not appear to be primary regulators of β_2_-adrenoceptor synthesis. A possible explanation was that Ang II acts on several different components of extracellular matrix formation and deposition to influence matrix turnover. Other beneficial effects of the angiotensin receptor blocker include lowering blood pressure and improving vascular function [27]. Taken together, losartan may prevent degradation of the β_2_-adrenoceptor to offer neuroprotective benefits.

In WKYs, pretreatment with Ang II pretreatment mimicked the SHR phenotype by inhibiting nicotine- and isoproterenol-induced BA relaxation (Figure 3). Ang II has also been shown to be closely associated with age-induced vascular dysfunction [28], impaired baroreceptor reflex function in the brainstem [29], elevated sympathetic nerve activity [30], and release of catecholamine from the C1 neuron of SHRs [31]. These findings suggest that Ang II may facilitate norepinephrine from the sympathetic nerve terminals. On the contrary, Ang II inhibited the axo-axonal interactions that elicited nitrergic vasodilation of BA in WKYs.

The inhibitory effect of Ang II was reversed by marimastat and catalase (Figure 4). Ang II is known to enhance ROS production in the nucleus tractus solitarius [32] and impair functional hyperemia via AT1 receptors in neocortical arterioles [10]. Furthermore, Ang II-mediated MMP activation and subsequent β_2_-adrenoceptor cleavage appear to be driven by NF-κB signaling. Since NF-κB inhibitors block receptor cleavage [33] and losartan inhibit Ang II-induced NF-κB activity [34], losartan effectively counteracts the Ang II/NF-κB/MMP/ROS pathway. By preserving β_2_-adrenoceptor density and restoring axo-axonal-elicited nitrergic vasodilation, losartan may mitigate the increased arteriolar tone characteristic of SHRs [33,35,36]. This mechanism likely contributes to the mitigation of neuroinflammation and cognitive decline.

Ang II inhibited nicotine-induced relaxation, and that inhibitory effect was reversed by ferrostatin-1 (Figure 4C). Ferroptosis is a newly discovered type of cell death triggered by intracellular phospholipid peroxidation [37]. Ferrostatin-1 has been shown to alleviate Ang II-induced inflammation and ferroptosis by suppressing ROS levels [14]. This suggests that ferroptosis may be involved in neuroinflammation and cognitive decline in hypertension, recognized as risk factors for Alzheimer’s.

While Ang II is known to stimulate ROS production in vascular smooth muscle cells (VSMCs) via NADPH oxidase [38], our findings suggest that the functional impairment leading to suppressed nitrergic dilation is primarily localized to parasympathetic nerve terminals. Specifically, although neurogenic vasorelaxation was abolished in SHRs, the vasodilatory response to isoproterenol, which acts directly on the smooth muscle’s β-adrenergic machinery, remained detectable and was restored by losartan. This functional dissociation indicates that the intrinsic relaxant capacity of the VSMCs remains operational, whereas the neurogenic signaling pathway, dependent on neurotransmitter release from nitrergic terminals, is selectively compromised. Furthermore, the robust restoration of nitrergic relaxation by Ferrostatin-1 provides strong functional evidence that the NOX/ROS/ferroptosis axis is the critical driver of the observed neurogenic deficit.

We acknowledge that while Ferrostatin-1 provided significant protection, pharmacological inhibition alone is insufficient to definitively establish ferroptosis as the dominant mechanism. A limitation of the current work is the lack of direct biochemical validation; therefore, future studies should incorporate additional measurements, such as lipid peroxidation levels, GPX4 and ACSL4 expression, and intracellular iron content, to further confirm the central role of ferroptosis in this context. Moreover, further investigation is needed to dissect the specific contributions of Nox isoforms using pharmacological inhibitors (such as VAS2870) or genetic models to fully elucidate the spatial specificity of oxidative stress within the neurovascular unit.

## 5. Conclusions

In summary, Ang II triggers ferroptosis within parasympathetic nerve terminals, leading to a functional imbalance between sympathetic and parasympathetic influences that impair neurogenic nitrergic dilation in the BAs of SHRs. Importantly, the inhibition of nicotine-induced relaxation by Ang II was reversed by ferrostatin-1 or losartan. These alterations in neurovascular signaling patterns suggest that targeting Ang II-induced ferroptosis may alleviate the cognitive decline associated with hypertension-related cerebrovascular dysfunction.

## Data Availability

The original contributions presented in this study are included in the article. Further inquiries can be directed to the corresponding author.

## Supplementary Materials

The following supporting information can be downloaded at: https://www.mdpi.com/article/10.3390/biomedicines14040853/s1, Figure S1. Baseline characteristics and hemodynamic effects of Losartan.

## Abbreviations

The following abbreviations are used in this manuscript:

BA	Basilar Artery
SHR	Spontaneously Hypertensive Rat
Ang II	Angiotensin II
WKY	Wistar-Kyoto rat
TNS	Transmural Nerve Stimulation
RHR	Renovascular Hypertensive Rats
BABF	Basilar Arterial Blood Flow
ROS	Reactive Oxygen Species
MMP	Metalloproteinase
SBP	Systolic Blood Pressure
SNP	Sodium Nitroprusside
AT1	Angiotensin II Type 1 Receptor
SCG	Superior Cervical Ganglion
