# Supplementary material for: Angiotensin II Disrupts Axo-Axonal Interaction-Mediated Vasorelaxation in Basilar Arteries of Normotensive and Hypertensive Rats
Source: Biomedicines. 2026 Apr 8;14(4):853. doi: 10.3390/biomedicines14040853 (PMC13113935; doi:10.3390/biomedicines14040853)
Supplement: Supplementary file 1 [file biomedicines-14-00853-s001.zip › biomedicines-4163836-supplementary.pdf]

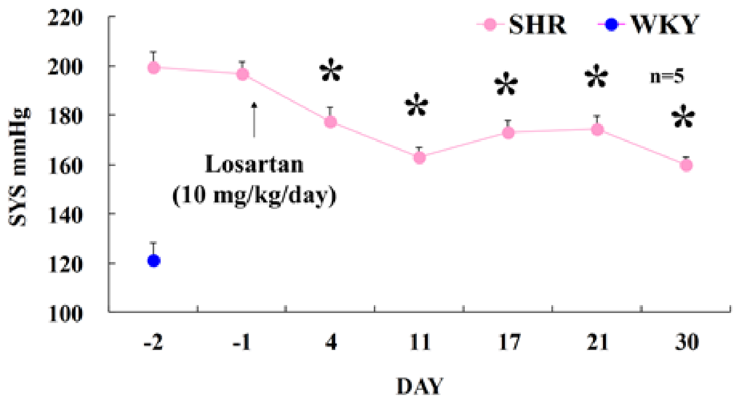

Figure S1. Baseline characteristics and hemodynamic effects of Losartan.

As shown in Supplemental Figure S1, the systolic blood pressure (SBP) was measured by the non-invasive tail-cuff method. 15–20-week-old SHRs exhibited significantly higher baseline SBP compared to age-matched WKY controls (199.4 ± 6.2 mmHg vs. 120.5 ± 7.4 mmHg, respectively; $p<0.05$). Chronic oral administration of losartan (10 mg/kg/day) progressively reduced SBP in the SHRs, with significant reductions observed starting from Day 4 (177.3 ± 5.8 mmHg) through to Day 30 (160 ± 2.9 mmHg). While losartan treatment effectively lowered blood pressure, these animals remained hypertensive relative to the normotensive WKY group, consistent with the partial rescue observed in our functional studies.
